# Long non-coding RNA NRAV enhances proliferation and invasion of hepatocellular carcinoma cells by modulating the Wnt/β-catenin signaling pathway

**DOI:** 10.1080/21655979.2022.2062977

**Published:** 2022-04-18

**Authors:** Qingxian Wang, Yumei Tang, Yuansen Ge, Songming Zhang, Meiyuan Zheng

**Affiliations:** a Department of Oncology, Tangshan City Hospital of Traditional Chinese Medicine, Hebei, China; b Internal Medicine of Chinese Medicine, North China University of Science and Technology, Hebei, China

**Keywords:** lncRNA, NRAV, miR-199a-3p, CISD2, wnt/β-catenin, HCC

## Abstract

Many dysregulated lncRNAs have been reported to perform an integral function in hepatocellular carcinoma (HCC). However, the role of long non-coding RNA (lncRNA) NRAV in HCC has not been elucidated. To address this issue, we investigated the function of NRAV in HCC in this research. Through bioinformatics prediction and real-time quantitative polymerase chain reaction validation, we found that NRAV plays an upmodulating role in HCC cells and tissues, and patients with high NRAV expression showed a poor prognosis. Cell viability was examined by conducting a Cell Counting Kit-8 analysis. Subsequently, the proliferation capacity of the cells was analyzed utilizing cell colony formation assay, and transwell invasion experiments were conducted to identify the cell invasion ability. To determine the association between NRAV and miR-199a-3p, and CDGSH iron-sulfur domain-containing protein 2 (CISD2), we conducted a dual luciferase assay. The protein and gene expressions were estimated utilizing Western blot. Findings illustrated that the overexpression of NRAV enhanced the HCC cell viability, proliferation and invasion, whereas they were inhibited significantly by down expression of NRAV. The dual-luciferase assay showed that miR-199a-3p is not only a target for NRAV but also interacts with the 3' UTR of CISD2 in HCC cells. MiR-199a-3p/CISD2 axis performs a function in NRAV-mediated cell behavior regulation. NRAV may trigger the Wnt/β-catenin signaling via the modulation of the miR-199a-3p/CISD2 axis in HCC. The findings of this work can provide novel insights into clinical diagnosis and the treatment of HCC in the future.

**Abbreviations:** HCC, hepatocellular carcinoma; LncRNA, long non-coding RNA; CISD2, CDGSH iron-sulfur domain-containing protein 2; CCK-8, Cell Counting Kit-8; cDNA, single‐stranded complementary DNA; RT-qPCR, real-time quantitative polymerase chain reaction; BCA, bicinchoninic acid; ceRNA, competing endogenous RNAs.

## GRAPHICAL ABSTRACT

**Figure:**
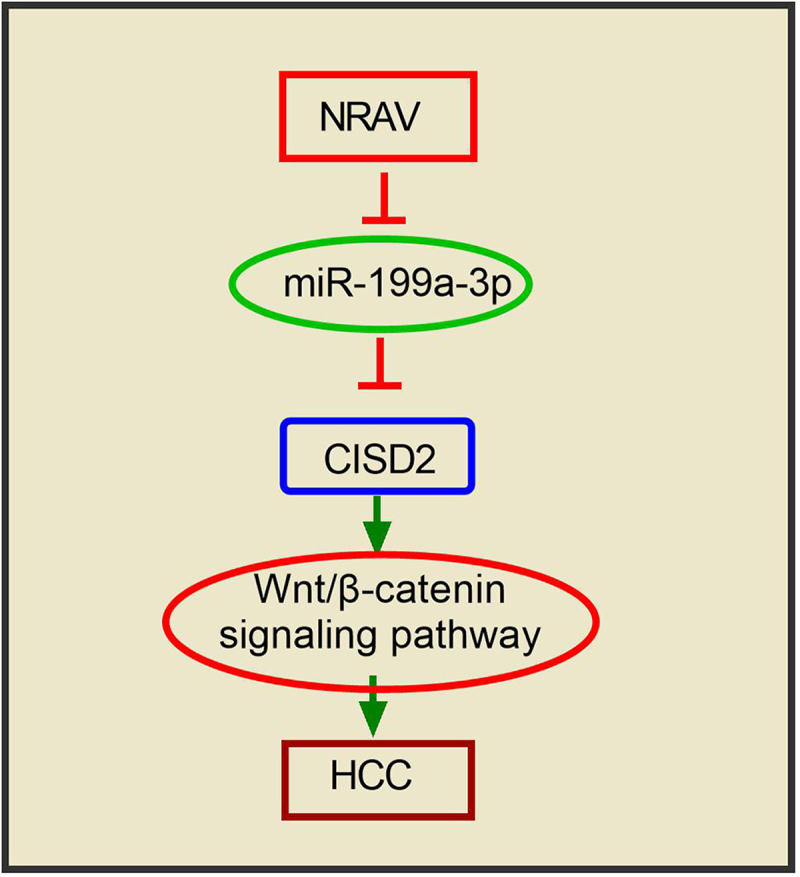

## Article highlights

NRAV was upregulated in HCC cells and tissues.

NRAV promoted the cell proliferation and metastasis of HCC cells.

NRAV activated the Wnt/β-catenin pathway via the miR-199a-3p/CISD2 axis.

## Introduction

Hepatocellular carcinoma (HCC) is among the most prevalent malignancies of the digestive tract and has been listed as the fourth most common malignancy and the third major contributor to cancer-associated mortality in the world [1,2]. Despite the significant progress made in HCC treatment and the development of innovative therapeutic strategies, surgery remains the preferred treatment option for HCC [3–6]. However, HCC patients have a dismal survival rate because of the high recurrence rate and metastasis after surgery [7]. Therefore, the pathogenesis of HCC for screening practical diagnostic markers and therapeutic HCC targets needs to be urgently examined.

Long non-coding RNAs (lncRNAs) are a class of RNA transcripts whose length exceeds 200 nucleotide non-protein RNAs [8–10]. Accumulating research evidence has indicated that lncRNAs can regulate the transcription and post-transcription of protein-coding genes and that they play an essential role in biological processes [10–12]. The lncRNAs play a crucial role in cancer research because they involve many aspects of tumor physical activity. Moreover, they are involved in several pathways through interactions with miRNAs or mRNAs in human cancers [13–16]. Many dysregulated lncRNAs have been identified by large-scale genomic screening in HCC, such as upregulated LINC01134, RHBN1-AS1, CMB9-22P13.1, MKLN1-AS, MAPKAPK5-AS1 and NRAV [17]. However, many of their biological functions and molecular mechanisms in HCC, especially those of NRAV, are not yet well understood. Current research suggests that the function of NRAV in HCC is related to immune checkpoints [18]. Other studies have shown that NRAV is a lncRNA associated with ferroptosis [19]. Interestingly, NRAV is involved in the replication of respiratory viruses and can even regulate the antiviral response of various viruses [20,21]. However, its function in HCC remains unclear.

This study will investigate the potential of lncRNA NRAV to regulate the miR-199a-3p/CISD2 axis and thus activate Wnt/β-catenin signaling in HCC. Our results show that lncRNA NRAV performs a crucial function in the occurrence and progression of HCC, thereby providing a new direction for HCC pathogenesis, clinical diagnostics or therapy.

## Materials and methods

### Tissue specimens

HCC specimens (n = 30) and adjoining non-tumor specimens (n = 30) were acquired from Tangshan City Hospital of Traditional Chinese Medicine from February 2016 to August 2018. The collected liver cancer tissues and adjacent tissues were surgically resected, and the tumor tissues and corresponding adjacent tissues (≥3 cm) were collected. All tumor tissues in the study were pathologically confirmed. The patients’ consent was then acquired, with the authorization of the ethics committee of Tangshan City Hospital of Traditional Chinese Medicine (approval *no*. ts201932511). HCC patients who underwent pre-operative anti-tumor therapy were excluded from the experiment. After excision, the tissue was frozen in liquid nitrogen before it was assessed by real-time fluorescence quantitative polymerase chain reaction (RT-qPCR).

### Cell lines and cell culture

HepG2, hepG2.2.15, hep3B, huh7 and normal human hepaRG were acquired from the American-Type Culture Collection (Manassas, VA, USA). The cells were fixed in Dulbecco’s modified Eagle’s medium (Gibco, Carlsbad, CA, USA) with 10% fetal bovine serum (FBS; Gibco, Carlsbad, CA, USA), 100 mg/mL streptomycin and 100 U/mL penicillin.

### RNA isolation and RT-qPCR

The total RNA from the cells and tissues was isolated by utilizing the TRIzol reagent. BeyoRT™ III cDNA synthesis kit (Beyotime Biotechnology, CAS# D7180L) was used for RNA reverse transcription. Genomic DNA was removed from the RNA samples, and then 6 μL DEPC-treated water and 4 μL BeyoRT™ III cDNA First Strand Premix (5X) were added to each tube, creating a total volume of 20 μL. The samples were mixed gently, and then the settling liquid was centrifuged. The samples were then incubated at 42°C for 10 min. The reverse transcriptase (which can also deactivate gDNA EZeraser) was inactivated, and the reverse transcription reaction was terminated. The reverse transcription products can be used directly for subsequent PCR reactions or frozen at −20°C for future use. The q-PCR was conducted in an Applied Biosystems 7500 real-time PCR system utilizing a BeyoFast™ SYBR Green qPCR mix (Beyotime Biotechnology, CAS# D7260). The 2^−ΔCt^ or 2^−ΔΔCt^ method was utilized to examine the levels of CISD2, miR-199a-3p and lncRNA NRAV expression. GAPDH can be used as an internal reference for quantitative NRAV and CICD2, and U6 was used as an internal reference for quantitative miR-199a-3p. The following primers were used: NRAV: 5'- GGAGTTGATGCCTCCGAACA-3' (Forward) and 5'- ATGACCGGAGCTGAAAGGTG-3' (Reverse); CISD2: 5'- CATTACCGGGTTCGCTAGGC-3' (Forward) and 5'- GTTTTAGAACGCCAACACCTACA-3' (Reverse); GAPDH: 5'- GGAGCGAGATCCCTCCAAAAT-3' (Forward) and 5'- GGCTGTTGTCATACTTCTCATGG-3' (Reverse); miR-199a-3p: 5'-TGCGGACAGTAGTCTGCACATT-3' and 5'-CCAGTGCAGGGTCCGAGGT-3' (Reverse); U6: 5'-TGCGGGTGCTCGCTTCGGCAGC-3' (Forward) and 5'-CCAGTGCAGGGTCCGAGGT-3' (Reverse).

### Cell transfection

Lipofectamine 3000 was utilized to transfect plasmids and miRNAs into HCC cells (Invitrogen, Carlsbad, CA, USA).

### CCK-8 assay

The transfected cells (2,000 cells per well) were cultivated in 96 wells for 24, 48, 72 and 96 h, respectively. Each well received 10 μL of CCK-8 solution (Biosharp, China), which was then incubated for an additional 2 h at a temperature of 37°C for 2 h. Lastly, we evaluated the absorbance wavelength at 450 nm by using a microplate reader.

### Colony formation assay

Upon inoculation of the transfected cells into 12-well plates (300 cells/well), the culture media was changed once after every three days. After 14 days, the cells were subjected to crystal violet staining, and the colonies comprising 50 or more cells were selected and photographed.

### Transwell assay

The transwell assay was conducted in accordance with previous literature [22]. The invasion abilities of HCC cells were transfected with the specified oligonucleotides or plasmids for 24 hours. Then, the transfected HCC cells were introduced to the top chamber (2 × 10^4^ cells), which contained 30 μL of Matrigel (1 mg/mL). Next, the bottom chambers were loaded with 600 μL of the complete medium that contains 20% FBS, and the culture process was maintained for another 48 h. The membranes were fixed for 20 minutes using 4% paraformaldehyde, followed by staining with 0.5% crystal violet. A microscope was used to count the number of cells in each field, and photographs were taken.

### Dual-luciferase reporter assay

The dual-luciferase reporter tests were conducted in accordance with the literature, with minor changes [23]. To verify the relation between miR-199a-3p and NRAV, co-transfection of the pGL3 basic vector carrying the mutant (MUT) or wild type (WT) sites of miR-199a-3p on NRAV was performed utilizing miR-NC, inhibitor-NC, inhibitor-miR-199a-3p and miR-199a-3p mimics into HCC cells. Similarly, to validate the relation between CISD2 and miR-199a-3p, co-transfection of the pGL3 basic vector carrying the WT or MUT sites of miR-199a-3p on the 3' UTR of CISD2 was conducted utilizing miR-NC, inhibitor-NC, inhibitor-miR-199a-3p and miR-199a-3p mimics into HCC cells. A dual luciferase assay kit was employed to evaluate the activity of luciferase after 48 h (Promega, USA).

### Western blot analysis

RIPA reagent (Sigma, USA) was employed for total protein isolation from HCC cell specimens, and the BCA Protein Assay Kit (Biosharp, China) was used to determine the protein levels. Then, with the addition of 30 μg of the sample to the loading hole, the proteins were separated utilizing 10% SDS-PAGE and then loaded onto PVDF membranes (0.45 μm, Merck Millipore). After blockage by 5% nonfat milk incubation to reduce noise, the membranes were immersed in the primary antibodies, which were diluted by the primary antibody diluent (Biosharp, China). The following primary antibodies were used: CISD2 (Rabbit, 1:1000, CAS #60,758, CST, USA), β-catenin (Rabbit, 1:1000, CAS # 8480, CST, USA), p-β-catenin (Rabbit, 1:2000, CAS # 9561, CST, USA), GSK3B (Rabbit, 1:1000, CAS #12,456, CST, USA), p-GSK3B (Rabbit, 1:1000, CAS #5558, CST, USA) and GAPDH (Rabbit, 1:5000, CAS #10,494-1-AP, Proteintech, China). The membranes were washed using PBS, and then they were probed with HRP-labeled secondary antibodies. The signals were visualized using the ECL reagent (Biosharp, China).

### Statistical analysis

All results are expressed as mean ± SD from a minimum of 3 separate experiments or replications. The statistical analyses were conducted by using GraphPad Prism 7.0 (GraphPad Software, La Jolla, CA, USA). Student’s t-test was performed to determine whether any variations existed between the cohorts, and a one-way analysis of variance accompanied by Tukey post-hoc test was employed to determine whether any variations existed between numerous cohorts. A p-value <0.05 was deemed statistically significant.

## Results

### NRAV was abnormally upmodulated in HCC cells and tissues

**Figure 1.**
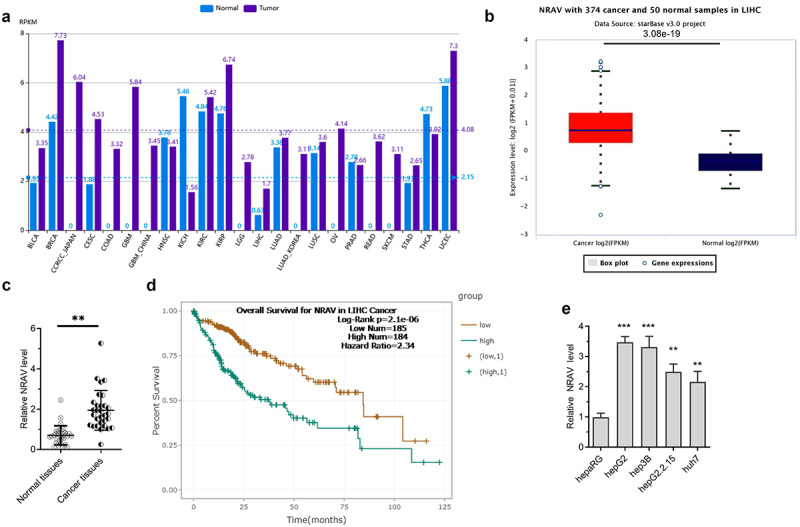
**NRAV was evidently upmodulated in HCC cells and tissues**. (a) The expression of NRAV was analyzed by lncRNASNP2 (http://bioinfo.life.hust.edu.cn/lncRNASNP#!/lncrna_info?lncrna=NONHSAT031176.2) in 23 tumor types. (b) ENCORI (The Encyclopedia of RNA Interactomes, https://starbase.sysu.edu.cn/) was utilized to anticipate the NRAV expression in HCC tissues. (c) RT-qPCR was performed to verify the NRAV expression in normal tissues (n = 30) and HCC tissues (n = 30), 2^−ΔCt^ method. (d) ENCORI was applied to anticipate the HCC patients’ survival curves according to the NRAV expression. (e) HCC cells and normal hepaRG cells were subjected to RT-qPCR to verify the expression of NRAV (hepG2, hepG2.2.15, hep3B and huh7), 2^−ΔΔCt^ method. **p < 0.01, ***p < 0.001.

We estimated the NRAV expression in HCC patients by firstly analyzing it using the online tool lncRNASNP2 [24]. The results indicated that NRAV was upregulated in various cancers, including liver hepatocellular carcinoma (LIHC) (Figure 1a). Then, we confirmed the results by using another online tool called ENCORI [25]; the same result as that predicted through lncRNASNP2 in LIHC was obtained (Figure 1b). We further demonstrated the results through RT-qPCR in normal tissues (n = 30) and HCC tissues (n = 30). The results were coincident, thus indicating that NRAV was significantly upregulated in LIHC (Figure 1c). The high expression of NRAV in the patients with LIHC indicated a poor prognosis (Figure 1d). In addition, we detected the NRAV expression in normal hepaRG and HCC cells (hepG2.2.15, hepG2, hep3B and huh7) and found that NRAV was upmodulated in HCC cell lines, unlike in normal hepaRG cells (Figure 1e). The above findings illustrated that NRAV was upmodulated in HCC cells and tissues and that an elevated NRAV expression is correlated with HCC progression.

### NRAV effectively enhanced HCC cell proliferation and invasion

**Figure 2.**
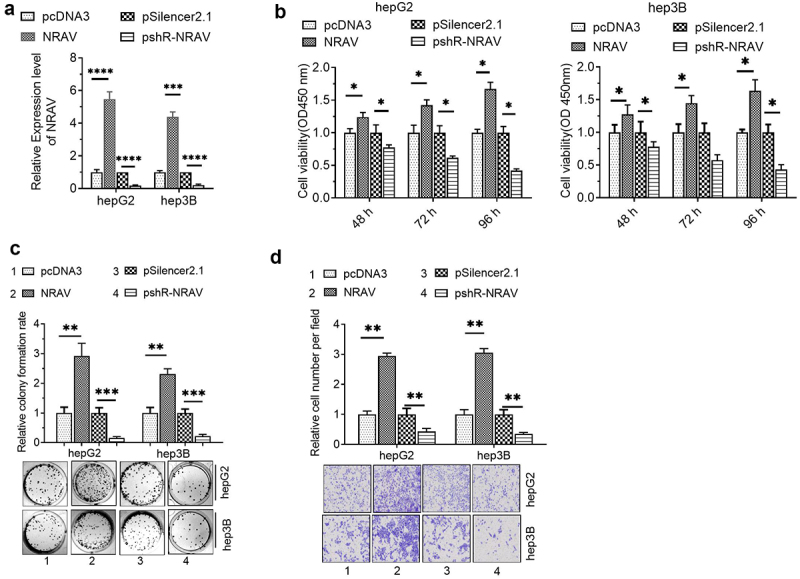
**NRAV effectively enhanced the proliferation and invasion of HCC cells**. (a) RT-qPCR was utilized to validate the NRAV profile in HCC cells after transfection, 2^−ΔΔCt^ method. (b) CCK-8 assay was employed to assess the HCC cell viability. (c–d) Effect of NRAV on the HCC cell proliferation and invasion. *p < 0.05, **p < 0.01, ***p < 0.001, ****p < 0.0001.

To investigate the role of NRAV in HCC cells, we firstly detected the efficiency of NRAV overexpression or knockdown plasmids by utilizing RT-qPCR. The results illustrated that transfection with overexpressed NRAV and plasmid knockdown significantly regulated the NRAV expression in hepG2 and hep3B cells (Figure 2a). Functionally, the CCK-8 assay demonstrated that overexpressed NRAV significantly improved the HCC cell viability, whereas the NRAV down expressiondecreased the viability significantly (Figure 2b). The cell colony formation experiment showed that the overexpressed NRAV strongly enhanced the proliferation of HCC cells, whereas its down expression weakened the cell proliferation abilities significantly (Figure 2c). The findings of the transwell invasion experiment demonstrated that the NRAV overexpression greatly increased the HCC cell invasion ability, whereas the down expression significantly inhibited the invasion ability (Figure 2d). These results showed that NRAV overexpression effectively increased the HCC cell proliferation and invasion, whereas NRAV down expression substantially impeded the cell proliferation and invasion capacities.

### MiR-199a-3p bound to NRAV in HCC cells

**Figure 3.**
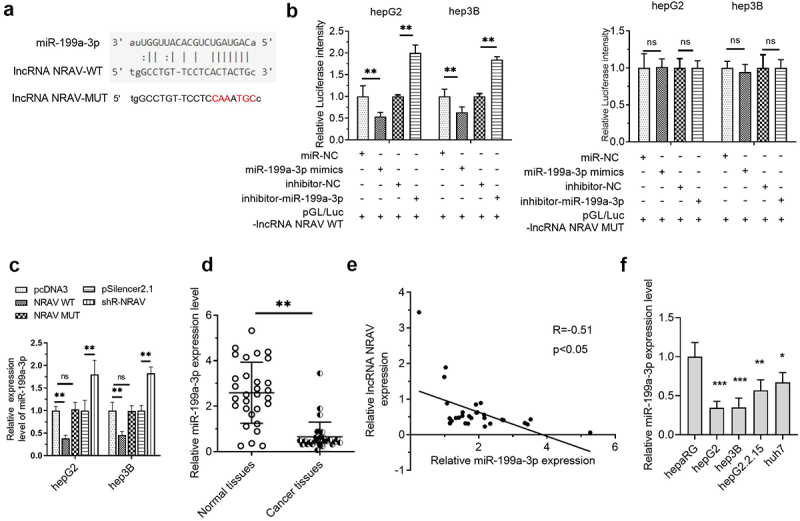
**NRAV was targeted by miR-199a-3p in HCC cells**. (a) LncRNASNP2 (http://bioinfo.life.hust.edu.cn/lncRNASNP#!/lncrna_info?lncrna=NONHSAT031176.2) was used to predict the binding of miRNAs on NRAV, and the mutant NRAV was indicated. (b) The dual-luciferase assay was employed to validate the target relations between NRAV and miR-199a-3p. (c) RT-qPCR was employed to validate the effect of overexpressed/silenced NRAV on miR-199a-3p, 2^−ΔΔCt^ method. (d) RT-qPCR was utilized to estimate the miR-199a-3p expression in normal tissues (n = 30) and HCC tissues (n = 30), 2^−ΔCt^ method. (e) The expression association between NRAV and miR-199a-3p was examined utilizing Pearson’s correlation coefficient. (f) HCC cells and normal hepaRG cells were subjected to RT-qPCR to confirm the expression of miR-199a-3p (hepG2, hepG2.2.15, hep3B and huh7), 2^−ΔΔCt^ method. *p < 0.05, **p < 0.01, ***p < 0.001; ns, no significance.

In investigating the potential mechanism of NRAV in HCC, LncRNASNP2 was used to predict the binding of miRNAs on NRAV. The results illustrated that miR-199a-3p is a promising NRAV target (Figure 3a). Further studies showed that the luciferase activity was reduced upon co-transfection with NRAV-WT and miR-199a-3p mimics in HCC cells and that it was elevated upon co-transfection with NRAV-WT and miR-199a-3p inhibitor. However, no significant change was observed after co-transfection with NRAV-MUT (Figure 3b). Moreover, NRAV overexpression and knockdown reduced and elevated the expression of miR-199a-3p, respectively (Figure 3c). In addition, miR-199a-3p expression was reduced in HCC tissues as opposed to that in normal tissues (Figure 3d). Importantly, NRAV expression was negatively associated with miR-199a-3p in HCC tissues (Figure 3e). Finally, we found that miR-199a-3p was generally downregulated in HCC cell lines compared with that in normal hepaRG cells (figure 1f). These findings indicated that NRAV targeted miR-199a-3p in HCC.

### MiR-199a-3p interplayed with the 3' UTR of CISD2 in HCC cells

**Figure 4.**
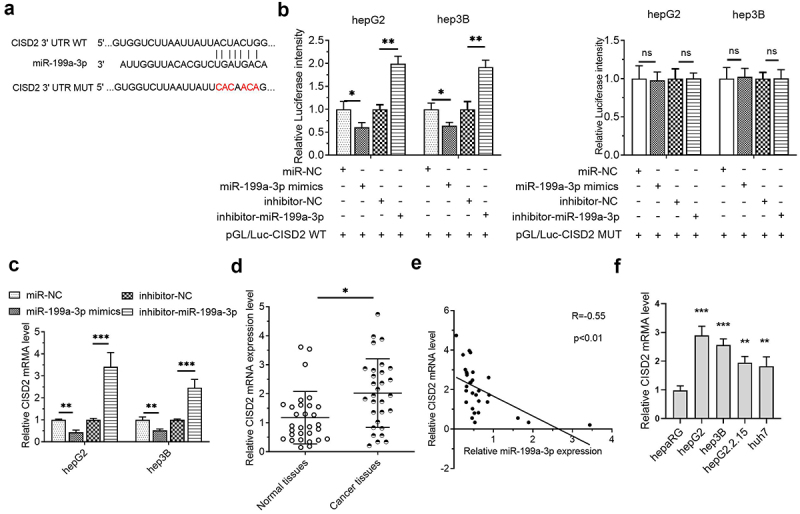
**CISD2 was a miR-199a-3p target in HCC cells**. (a) The miR-199a-3p targets were predicted utilizing TargetScan Human 7.2 (http://www.targetscan.org/vert 80/), and the CISD2 mutant 3' UTR was identified. (b) The target connections between miR-199a-3p and CISD2 were validated utilizing the dual luciferase assay. (c) RT-qPCR was utilized to validate the impacts of miR-199a-3p on the level of CISD2 mRNA, 2^−ΔΔCt^ method. (d) The CISD2 mRNA level was determined by RT-qPCR in normal (n = 30) and HCC (n = 30) tissues, 2^−ΔCt^ method. (e) The expression association between miR-199a-3p and CISD2 was analyzed using Pearson’s correlation coefficient. (f) HCC cells and normal hepaRG cells were subjected to RT-qPCR to confirm the mRNA of CISD2 (hepG2, hepG2.2.15, hep3B and huh7), 2^−ΔΔCt^ method. *p < 0.05, **p < 0.01, ***p < 0.001; ns, no significance.

Next, we investigated the downstream targets of miR-199a-3p. The results of the TargetScanHuman 7.2 online prediction tool illustrated that CISD2 was a possible miR-199a-3p target (Figure 4a). The luciferase activity was minimized upon co-transfection with CISD2-WT and miR-199a-3p mimics in HCC cells and was enhanced upon co-transfection with CISD2-WT and miR-199a-3p inhibitor. However, no significant change was detected after co-transfection with CISD2-MUT (Figure 4b). In addition, CISD2 mRNA levels in the miR-199a-3p mimic cohort were reduced. However, in the miR-199a-3p inhibitor cohort, CISD2 mRNA levels were increased (Figure 4c). CISD2 mRNA levels in HCC tissues were markedly increased in contrast to those in normal tissues (Figure 4d). Furthermore, the miR-199a-3p expression was negatively associated with CISD2 mRNA levels in HCC tissues (Figure 4e). Lastly, we found that CISD2 mRNA was generally upregulated in HCC cell lines compared with that in normal hepaRG cells (figure 1f). These results indicated that CISD2 is a miR-199a-3p target in HCC.

### MiR-199a-3p/CISD2-mediated NRAV function in HCC proliferation and invasion

**Figure 5.**
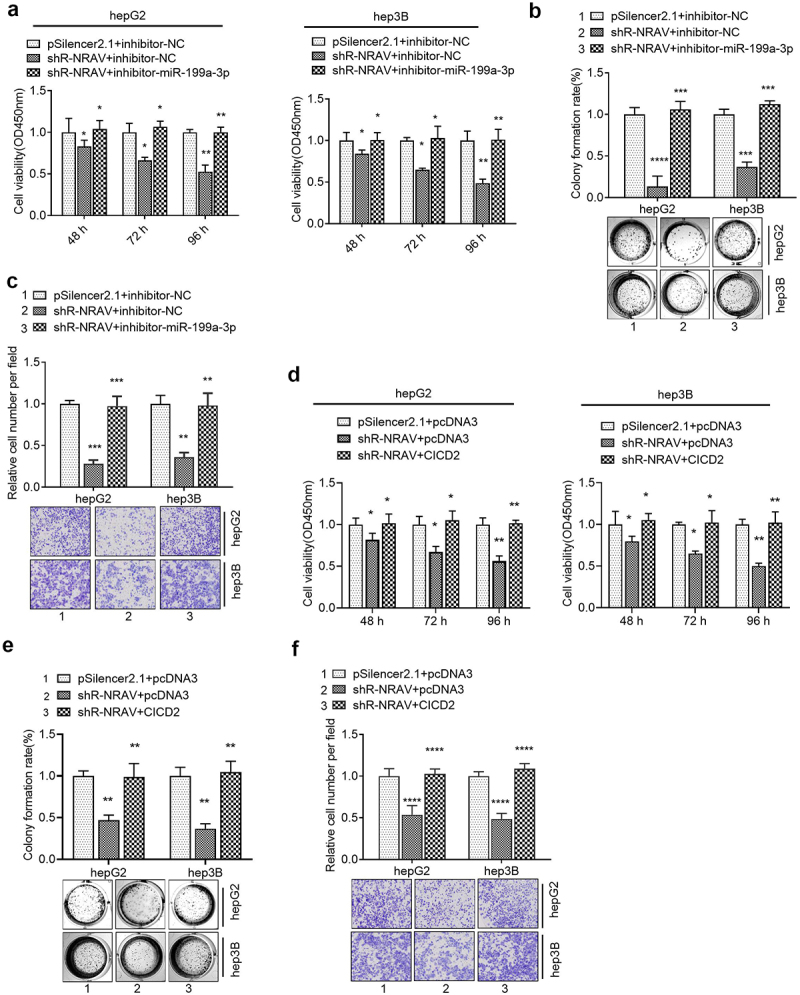
**Rescue experiments were used to verify that NRAV regulated the cell phenotype through miR-199a-3p/CICD2 axis**. (a–c) Effect of co-transfection of miR-199a-3p inhibitor and shR-NRAV on the HCC cell viabilities, proliferation and invasion. (d–f) Effect of co-transfection of miR-199a-3p inhibitor and shR-CICD2 on the HCC cell viabilities, proliferation and invasion. *p < 0.05, **p < 0.01, ***p < 0.001, ****p < 0.0001.

The rescue experiment was performed to verify whether NRAV regulates the malignant behavior of HCC cells through miR-199a-3p /CISD2 axis. Firstly, the knockdown of NRAV impeded HCC cell viability, proliferation and invasion, which was partly reversed by the co-transfection of miR-199a-3p inhibitors and sh-NRAV (Figures 5A–5c). In addition, the co-transfection of sh-NRAV and CISD2 overexpressing plasmids partly counteracted the inhibition induced by the transfection of sh-NRAV alone (Figures 5D–5e). These findings illustrated that the miR-199a-3p/CISD2 axis was involved in NRAV-mediated cell behavior regulation.

### NRAV activated Wnt/β-catenin signaling through miR-199a-3p/CISD2-mediated mechanism

**Figure 6.**
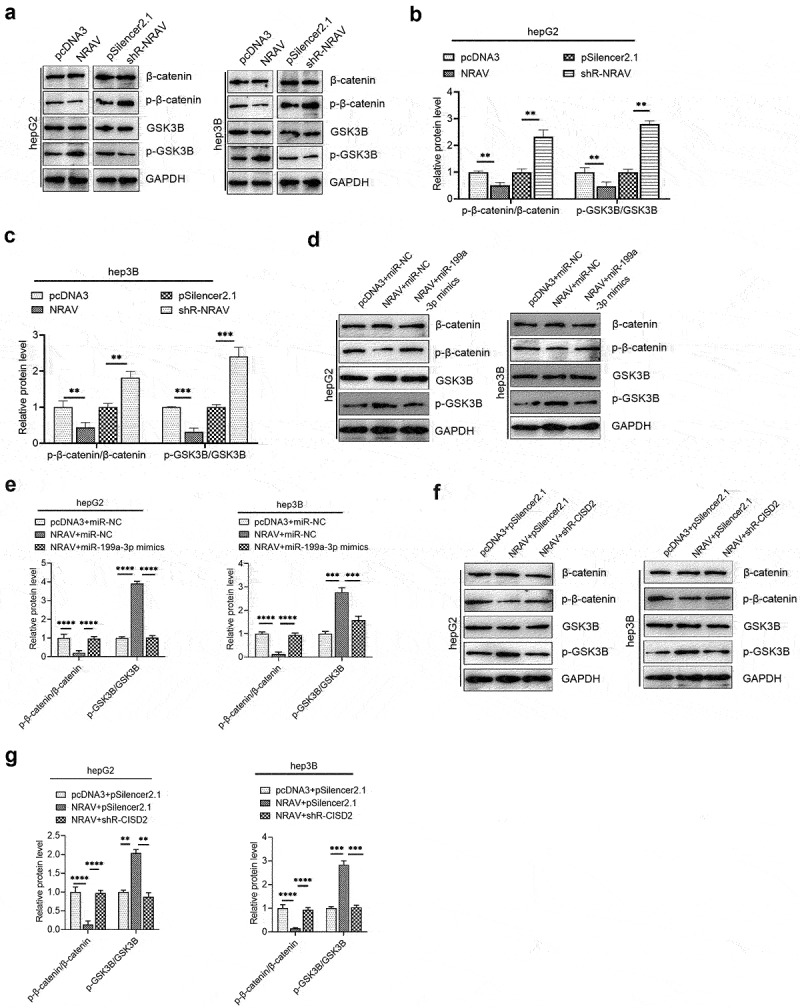
**NRAV regulated the Wnt/β-catenin pathway via miR-199a-3p/CICD2 axis**. (a) Western blot was employed to identify the protein levels of p-β-catenin, GSK3B, β-catenin and p-GSK3B by overexpression or knockdown of NRAV in HCC cells. (b and c) The p-GSK3B/GSK3B and p-β-catenin/β-catenin protein levels in (d) were quantified. Western blot of the p-β-catenin, β-catenin, p-GSK3B and GSK3B showed that protein levels were partly reversed by co-transfection using miR-199a-3p mimics. (e) The p-GSK3B/GSK3B and p-β-catenin/β-catenin protein levels in (c) were quantified. (f) Western blot of the p-β-catenin, β-catenin, p-GSK3B and GSK3B showed that protein levels were partly reversed by co-transfection with shR-CISD2. (g) Quantification of the p-GSK3B/GSK3B and p-β-catenin/β-catenin protein levels in (F). **p < 0.01, ***p < 0.001.

Increasing evidence has shown the oncogenic function of the Wnt/β-catenin pathway in several cancers [26]. According to recent reports, CISD2 can activate the Wnt/β-catenin pathway in pancreatic cells [27]. Therefore, we further tested whether NRAV can trigger the Wnt/β-catenin pathway via the CISD2/miR-199a-3p axis in HCC cells. We demonstrated that the overexpression of NRAV inhibits the β-catenin to p-β-catenin ratio in HCC cells, whereas the silencing of NRAV markedly enhances the p-β-catenin to β-catenin ratio (Figures 6A–6c). This finding indicates that β-catenin decomposed by the proteasome is reduced, thus stabilizing β-catenin and triggering the Wnt/β-catenin pathway. Furthermore, the p-GSK3B/GSK3B ratio increased when NRAV was overexpressed, and it decreased under the knockdown of NRAV (Figures 6A–6c). These results indicate that the active GSK3B, which could generate a detection site on β-catenin by phosphorylating a conserved Ser/Thr-rich series situated proximal to the amino-terminal, was reduced when NRAV was overexpressed. Moreover, NRAV overexpression inhibited the p-β-catenin to β-catenin ratio but elevated the p-GSK3B to GSK3B ratio in HCC cells, which was partly reversed by the co-transfection of NRAV overexpressing vector and miR-199a-3p mimics (Figures 6D and 6e). In addition, the co-transfection of the NRAV overexpressing vector and sh-CISD2 vector partly counteracted the ratios of p-GSK3 to GSK3B and p-β-catenin to β-catenin under the transfection of NRAV overexpressing vector alone (Figures 6f–6g). These results indicate that NRAV stimulated Wnt/β-catenin signaling via the miR-199a-3p/CISD2 axis in HCC.

## Discussion

Accumulating research evidence has demonstrated that non-coding RNAs, including miRNAs and lncRNAs, are important regulators in the progression of liver cancer and have promising diagnostic and treatment values [28,29]. The dysregulation of lncRNAs can lead to cancer. Thus, many tumor-related lncRNAs have been identified in HCC, and most of their molecular mechanisms have been well investigated. For example, LINC00174, lncTCF7 and lncRNA RMRP act as oncogenic lncRNAs in HCC [30–32]. However, to date, the role of lncRNA NRAV in HCC remains unclear.

So far, the role of NRAV has been rarely studied. Xu et al. indicated that NRAV has an immune-related lncRNA signature in HCC and might be related to immune checkpoints [18]. Chen et al. showed that NRAV is a ferroptosis-related lncRNA in HCC [19]. Note that NRAV might be involved in the regulation of virus replication and host antiviral responses [20,21]. This research focused on investigating the function and underlying mechanism of lncRNA NRAV in HCC cells. We illustrated that lncRNA NRAV was upmodulated and miR-199a-3p was downmodulated in HCC. Moreover, miR-199a-3p was a target of NRAV, which could promote HCC progression through the suppression of the miR-199a-3p expression level. miR-199a-3p certainly played a role in cancers, especially in HCC. As a key member of the miR-199 family, miR-199a-3p functions as a tumor repressor in diverse tumors. The elevated expression of miR-199a-3p might directly target CD44, thereby inhibiting the proliferation of HCC cells [33]. In addition, miR-199a-3p suppressed the progression of HCC cells by modulating other targets, such as ZHX1 or PUMA [34]. Our findings illustrated that the miR-199a-3p expression in HCC cells and tissues was decreased compared with the normal tissues and that NRAV was inversely associated with that of miR-199a-3p in HCC tissues, thereby additionally confirming the tumor repressor function of miR-199a-3p in HCC.

In general, lncRNAs regulate gene expression by serving as ceRNAs, and we demonstrated that in HCC, NRAV can competitively bind to miR-199a-3p. We further discovered that miR-199a-3p targeted the 3' UTR of CISD2 and inhibited its expression. CISD2 was upmodulated in HCC tissues, and CISD2 mRNA levels were inversely associated with the miR-199a-3p expression. Further rescue experiments proved that NRAV regulated the proliferation and the invasion by miR-199a-3p/CISD2 axis-mediated mechanism in HCC.

CISD2 is an evolutionarily highly conserved protein that performs an integral function in aging-related diseases and cancers, including neuronal loss, cell drug resistance, cell proliferation, migration and invasion [27,35,36]. Yang et al. reported that CISD2 promotes proliferation and EMT in pancreatic cancer cells by stimulating the Wnt/β-catenin pathway [27]. In addition, miR-199a-3p can target Wnt/β-catenin in human diseases [37–39]. The Wnt/β-catenin cascade is a modulator of cell fate and performs an oncogenetic function in cancers [40–42]. This work is the first to demonstrate that NRAV stimulated Wnt/β-catenin signaling via the miR-199a-3p/CISD2 axis in HCC.

## Conclusion

NRAV was upregulated in HCC and may enhance cell proliferation and invasion by inhibiting miR-199a-3p, thereby increasing the CISD2 expression. In addition, NRAV could activate Wnt/β-catenin signaling through the miR-199a-3p/CISD2 axis in HCC. The results of this work will contribute to clinical diagnosis or facilitate the development of effective therapeutic measures against HCC.

## Data Availability

Please contact the corresponding author to request data.
